# Tumoral Neuroligin 1 Promotes Cancer–Nerve Interactions and Synergizes with the Glial Cell Line-Derived Neurotrophic Factor

**DOI:** 10.3390/cells11020280

**Published:** 2022-01-14

**Authors:** Laura Bizzozero, Margherita Pergolizzi, Davide Pascal, Elena Maldi, Giulia Villari, Jessica Erriquez, Marco Volante, Guido Serini, Caterina Marchiò, Federico Bussolino, Marco Arese

**Affiliations:** 1 Department of Oncology, University of Torino, 10060 Candiolo, Italy; laura.bizzozero@unito.it (L.B.); margherita.pergolizzi@unito.it (M.P.); pascal.davide@gmail.com (D.P.); giulia.villari@ircc.it (G.V.); marco.volante@unito.it (M.V.); guido.serini@unito.it (G.S.); federico.bussolino@unito.it (F.B.); 2 Candiolo Cancer Institute, FPO-IRCCS, 10060 Candiolo, Italy; jessica.erriquez@ircc.it; 3 Pathology Unit, Candiolo Cancer Institute, FPO-IRCCS, 10060 Candiolo, Italy; elena.maldi@ircc.it (E.M.); caterina.marchio@ircc.it (C.M.); 4 Department of Medical Sciences, University of Turin, 10124 Turin, Italy

**Keywords:** tumor–nervous connections, neuroligin 1, glial cell line-derived neurotrophic factor, cofilin, filopodia

## Abstract

Many nervous proteins are expressed in cancer cells. In this report, we asked whether the synaptic protein neuroligin 1 (NLGN1) was expressed by prostatic and pancreatic carcinomas; in addition, given the tendency of these tumors to interact with nerves, we asked whether NLGN1 played a role in this process. Through immunohistochemistry on human tissue microarrays, we showed that NLGN1 is expressed by prostatic and pancreatic cancer tissues in discrete stages and tumor districts. Next, we performed in vitro and in vivo assays, demonstrating that NLGN1 promotes cancer cell invasion and migration along nerves. Because of the established role of the neurotrophic factor glial cell line-derived neurotrophic factor (GDNF) in tumor–nerve interactions, we assessed a potential NLGN1–GDNF cooperation. We found that blocking GDNF activity with a specific antibody completely inhibited NLGN1-induced in vitro cancer cell invasion of nerves. Finally, we demonstrated that, in the presence of NLGN1, GDNF markedly activates cofilin, a cytoskeletal regulatory protein, altering filopodia dynamics. In conclusion, our data further prove the existence of a molecular and functional cross-talk between the nervous system and cancer cells. NLGN1 was shown here to function along one of the most represented neurotrophic factors in the nerve microenvironment, possibly opening new therapeutic avenues.

## 1. Introduction

The family of Neuroligins is composed of transmembrane post-synaptic proteins of the central nervous system (CNS), that function in the fine-tuning of synaptic activity [1]. We have previously shown that a member of this family, neuroligin 1 (NLGN1) transmembrane protein, is expressed in endothelial cells and modulates angiogenesis [2,3,4]. Here, we investigate instead the potential pro-tumoral activities of NLGN1 with a specific focus on the relation that cancer cells take with nerves in order to grow and spread (a general phenomenon that we call tumor–nervous connections or TNCs) [5]. TNCs include a wide range of processes, from direct physical interactions, such as in perineural invasion (PNI, i.e., the direct invasion of the nerve sheath by cancer cells) [6] to humoral interactions at the perineural niche (i.e., the microenvironment in the space surrounding a nerve) [7]. In the latter context, before tumor cells and nerves take direct contact, reciprocal autocrine and paracrine signaling takes place through a large group of growth factors, cytokines, and neurotransmitters. Among these, glial cell line-derived neurotrophic factor (GDNF) promotes migration and recruitment to the perineural microenvironment of cancer cells [8]. We describe a functional interaction between NLGN1 and GDNF in promoting TNCs.

## 2. Materials and Methods

Additional materials and methods are available as Supplementary Materials.

### 2.1. Ex Vivo Co-Culture Model of Nerve Invasion

We followed the model described in [9], with some modifications. Dorsal root ganglia (DRG), excised from 6–8-week-old C57BL6 mice, were seeded individually in a 20 µL drop of growth factor-reduced Matrigel (Corning) and co-cultured with 5 × 10^4^ cancer cells placed in another adjacent and contiguous 30 µL drop. The Matrigel/DRG/cancer cells system was cultured in RPMI-1640, containing 10% FBS in 5% CO_2_ at 37 °C. The invasion rate was calculated by measuring the area of cells that were in direct contact with neurites with ImageJ.

For co-cultures in the presence of a blocking antibody against the GDNF family receptor α (anti GFRα1 AF560, 10 µg/mL, R&D Biosystem, Minneapolis, MN, USA), cells were seeded after 5 days, once the neurites outgrowth was established. For experiments with DRG-conditioned medium, DRG were cultured in Matrigel alone and the medium was collected after five days.

### 2.2. In Vivo Model of Murine Sciatic Nerve Invasion, In Vivo Imaging, and Nerve Immunohistochemistry

NOD/SCID mice were randomly divided into 4 groups (*n* = 6) as follows: (i) PC3 shCTR, (ii) PC3 shNLGN1, (iii) MIA PaCa-2 PLVX, and (iv) MIA PaCa-2 PezNLGN1. The right sciatic nerve was surgically exposed and a 300,000/3 µL cell suspension was microscopically injected into the perineum using a microliter syringe (Hamilton, Toledo, OH, USA, 10 µL, 33G). For analgesia, mice were treated with 5 mg/kg Rimadyl 12 h before surgery and daily. At the same time, the animals were given antibiotic therapy with Baytril (2.5% injectable solution, 5 mg/kg) for a few days. Mice were followed for recovery every day for 72 h and monitored for nerve function.

In vivo bioluminescence imaging was performed and analyzed using an IVIS imaging system Spectrum CT (PerkinElmer, Waltham, MA, USA). The bioluminescent signal was induced by i.p. injection of D-luciferin (150 mg/kg in PBS) 8 min before in vivo imaging.

At the experimental endpoint, murine sciatic nerves were dissected up and embedded in Tissue-Tek OCT (Electron Microscopy Sciences, Hatfield, PA, USA). Frozen blocks were serially sectioned longitudinally at 5 and 8 μm thickness, respectively, using a Cryostat microtome (Leica CM1950, Wetzlar, Germany). Sections were used for H&E and IHC staining with an anti-Cytokeratin antibody (sc-32329, Santa Cruz, Dallas, TX, USA).

### 2.3. Live Imaging

For live imaging, MIA PaCa-2 cells, plated in a µ-Slide 8 Well IbiTreat (Ibidi, Gräfelfing, Germany), were transiently transfected with the fluorescent-tagged cDNA pEGFPN1 LifeAct (pEGFP-C1 Lifeact-EGFP was a gift from Dyche Mullins (Addgene plasmid # 58,470; http://n2t.net/addgene:58470; accessed on 21 December 2021, RRID:Addgene_58470). Cells were analyzed by using a Leica TCS SP8 AOBS confocal microscope equipped with 2 HyD, PL APO 63×/1.4, NA immersion objective was employed. Images of 512 × 512 pixels were acquired at pixel size = 161.51–182.01 nm. Image acquisition was performed by adopting a laser power, gain, and offset settings that allowed maintaining pixel intensities (gray scale) within the 0–255 range and hence avoiding saturation. Movies to image the actin filopodia were acquired for 5 min, taking one frame every 2.5 s. In particular, we quantified the physical parameters (number, length, and density) of filopodia using the Fiji plugin Filoquant, as described in [10].

Filopodia dynamics, in terms of velocity and tip distance, were manually tracked using Fiji plugin TrackMate. We tracked from 5 to 10 filopodia/cell over the filopodia lifetime in at least 11 cells (up to 14).

### 2.4. Statistical Analysis

Upon verification of normal distribution, the statistical significance of raw data between the groups in each experiment was evaluated using the unpaired Student’s *t*-test or ANOVA, followed by Dunn’s post-test. Results are expressed as mean ± SE when derived from averaged experiments, or as mean ± SD when derived from several data points of one experiment. *n* represents the number of individual experiments. The asterisks (*, **, and ***) in figure panels refer to statistical probabilities (*p*) of <0.05, <0.01, and <0.001, respectively.

## 3. Results and Discussion

### 3.1. Neuroligin 1 Is Expressed in Prostatic and Pancreatic Cancer

**Figure 1:**
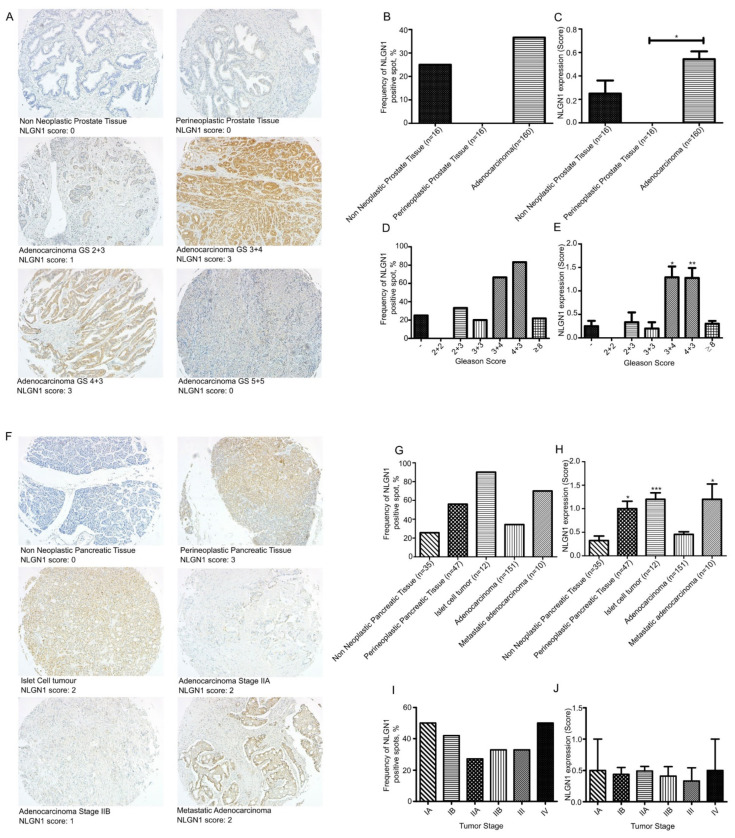
NLGN1 evaluation by IHC on a TMA of prostate adenocarcinoma (PR1921c—Biomax) (**A**–**E**) and two TMAs of pancreatic adenocarcinoma (PA961f and PA2081c—Biomax) (**F**–**J**). (**A**,**F**) Representative images of NLGN1 protein expression by IHC in TMA cores and their relative score (original magnification 10×). (**B**,**D**,**G**,**I**) Distribution of NLGN1 protein expression according to the IHC positivity (present or absent) in terms of (**B**,**G**) pathology diagnosis, (**D**) Gleason score (GS), and (**I**) tumor stage. (**C**,**E**,**H**,**J**) Distribution of NLGN1 protein expression according to the IHC score, as present (1, 2, 3) or absent (0), in terms of (**C**,**H**) pathology diagnosis, (**E**) Gleason score (GS), and (**J**) tumor stage. Values are mean ± SE; one-way ANOVA with Bonferroni test: *, *p* < 0.05 **, *p* < 0.01 ***, *p* < 0.001.

We investigated the expression of NLGN1 in prostatic (Figure 1A–E) and pancreatic (Figure 1F–J) cancers at the protein level by immunohistochemistry (IHC) on tissue microarrays (TMAs).

Among cores from non-neoplastic prostate samples, 4 out of 16 (25%) cores displayed NLGN1 expression, although at low intensity, while no perineoplastic tissues were expressed NLGN1 (Figure 1B). NLGN1 expression scores were significantly higher in adenocarcinomas (Figure 1C). None of the adenocarcinomas from Gleason score (GS) 2 + 2 showed positivity, while 2/6 (33%) carcinomas from GS 2 + 3, 2/10 (20%) from GS 3 + 3, 16/24 (66.6%) from GS 3 + 4, 15/18 (83.3%) from GS 4 + 3, and 21/96 (21.8%) from GS ≥ 8 showed homogeneous expression (Figure 1D). Finally, we observed significantly higher expression scores of NLGN1 in GS 3 + 4 and 4 + 3 (Figure 1E). This may suggest that the protein is transiently expressed in moderately differentiated prostatic cancer.

A total of 9 out of 35 (25.7%) cores from non-neoplastic pancreatic tissue, and 26/47 (55.3%) cores of perineoplastic pancreatic tissue showed NLGN1 expression (Figure 1G). Among neoplastic cores, 90% of islet cell tumors (10/12), 34.4% (52/151) of adenocarcinomas, and 70% (7/10) of metastatic adenocarcinoma expressed NLG1 (Figure 1G). NLGN1 protein expression displayed stronger intensity in perineoplastic tissues cores, and, interestingly, in neuroendocrine islet tumors and liver metastases (Figure 1H). Staging information were available and NLGN1-positive cores were present in: 1/2 (50%) stage IA, 15/55 (27.3%) stage IB, 29/68 (42.6%) stage IIA, 6/18 (33.3%) stage IIB, 2/6 (33.3%) stage III, and 1/2 (50%) stage IV (Figure 1I). Finally, among adenocarcinoma samples, we observed low scores of NLGN1 expression without a significant correlation with tumor differentiation (Figure 1J).

### 3.2. NLGN1 Promotes Tumor–Nerve Interactions

We next performed two assays to investigate the role of tumoral NLGN1 in TNCs: an ex vivo DRG nerve invasion assay and an in vivo mouse sciatic nerve invasion assay. We chose, as cellular models, the PC3 prostatic and MIA PaCa-2 pancreatic cell lines (Figure S1), which, respectively, represent tumor cells expressing high or almost undetectable levels of NLGN1, in agreement with our observations in the human TMA.

**Figure 2:**
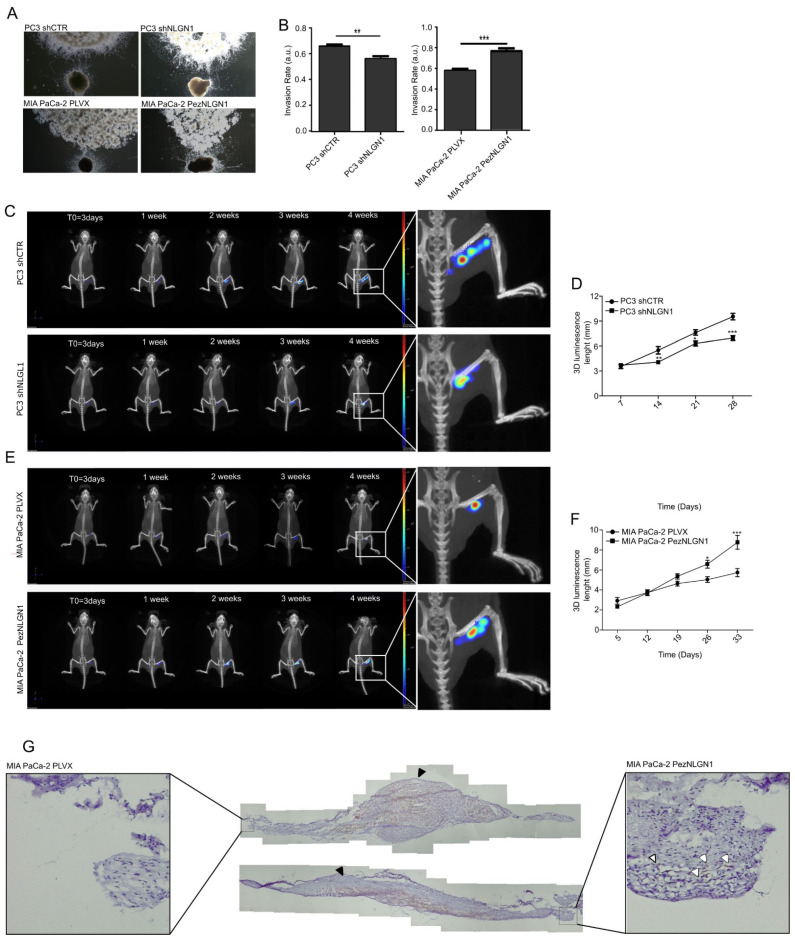
NLGN1 triggers cell invasion along the nerves in an ex vivo and in vivo models of perineural invasion. (**A**) Co-culture of murine DRG with prostate (PC3 shCTR or PC3 shNLGN1 cells, upper panels) and pancreatic cancer cells (MIA PaCa-2 PLVX or PezNLGN1 cells, lower panels). Pictures were taken at 48 h and are representative of 1 of 3 reproducible experiments performed in quadruplicate (original magnification 2.5×). (**B**) Quantification of the invasion rate. Values are mean ± SE of 3 independent experiments. Unpaired Student’s *t*-test, two tailed: **, *p* < 0.01, ***, *p* < 0.001. (**C**–**G**) 3 × 10^5^ PC3 shCTR and PC3 shNLGN1 (**C**,**D**), or MIA PaCa-2 PLVX and MIA PaCa-2 PezNLGN1 (**E**–**G**) cells, further infected with a CMV-Luc vector, were surgically inoculated into the right sciatic nerve of 6-week-old NOD/SCID mice. IVIS images shown in (**C**,**E**) are representative of 1 of 6 mice. Graphs in (**D**,**F**) show the evaluation of the length (mm) of the bioluminescence of tumor cells along the sciatic nerve over time. Values are expressed as mean ± SE, *n* = 6. Unpaired Student’s *t*-test, two tailed: *, *p* < 0.05 **, *p* < 0.01, ***, *p* < 0.001. (**G**) Cytokeratin staining (brown) by IHC on two sciatic nerves that were injected either with control or NLGN1-overespressing MIA PaCa-2 cells, and next surgically extracted at the endpoint. The cellular distribution of cells in the main tumor is visible (the site of tumor cell injection indicated by the black arrowheads), while distally migrating tumor cells (shown in the magnification by white arrowheads) are visible only with NLGN1-overexpressing MIA PaCa-2 cells.

The DRG assay (Figure 2A,B) demonstrated a positive correlation between NLGN1 expression and the extent of nerve invasion, with the NLGN1-expressing cells (wild type PC3 cells and NLGN1 overexpressing MIA PaCa-2 cells) migrating towards and along neurites at a faster rate than the NLGN1-null cells. The sciatic nerve assay demonstrated the same principle, whereby NLGN1 downregulation in PC3 cells (Figure 2C,D) or NLGN1 overexpression in MIA PaCa-2 cells (Figure 2E,F), induced, respectively, a decrease or an increase in cell migration along the nerve, measured as the length of the luminescent signal (magnifications in Figure 2C,E). Finally, IHC on surgically extracted nerves showed that only when overexpressing NLGN1 did the mass of cells within the nerve assume an elongated shape and migrate distally to the injection site, rather than forming an enlarged mass around the injection site, as observed in control cells (Figure 2G).

### 3.3. NLGN1 Enhances GDNF/GFRα1-Induced Migration and Invasion of Cancer Cells

A peculiar observation that we made on the human prostatic cancer TMA (Figure 1) was that at the stage in which NLGN1 is mostly expressed (GS 3 + 4 and 4 + 3), no cases of visible PNI were present. All PNI-positive cases were present at stages beyond these. Similarly, for the pancreatic cancer TMA, no significant relation between NLGN1 expression and PNI was detected. It appears as though, at stages when overt PNI is visible, NLGN1 expression has already fallen.

Nevertheless, cancer–nerve communication also occurs through soluble mediators before direct physical contacts are established and the nerve is invaded [7]. Since the DRG and sciatic nerve assays that we performed (Figure 2) clearly involved humoral cancer cells–nerve contacts in the early phases, NLGN1 could be playing a role in this context. One key soluble factor in the early events of tumor nerve communication is glial cell line-derived neurotrophic factor (GDNF) [8,11], which signals through the RET tyrosine kinase receptor and its glycosyl-phosphatidylinositol-anchored co-receptor, GFRα1. GDNF and GFRα1, released by nerves, activate their receptor expressed by cancer cells, inducing migration towards nerves [8].

**Figure 3:**
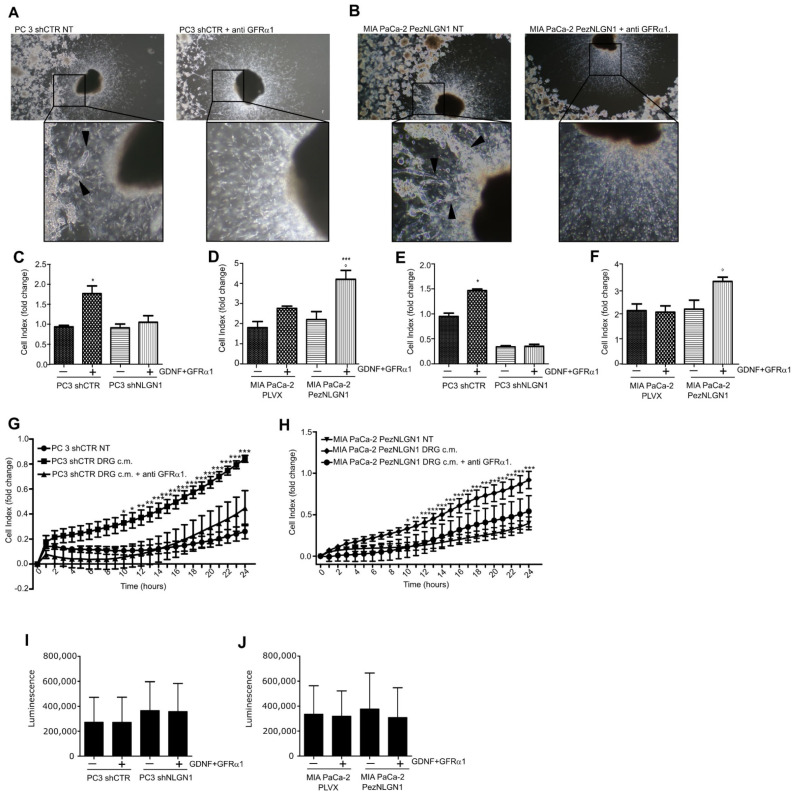
NLGN1 and GDNF act synergistically in tumor–nerve connections. (**A**,**B**) Co-culture of murine DRG with PC3 shCTR (**A**) or MIA PaCa2 PezNLGN1 (**B**) cells in absence or in presence of a blocking antibody against GFRα1. Black arrowheads in magnifications indicate PC3 shCTR (**A**) and MIA PaCa-2 PezNLGN1 (**B**) cancer cells contacting and migrating along the neurites toward the DRG body in absence of the GFRα1-blocking antibody. In the presence of GFRα1-blocking antibody, neurites appear free of invading cells (right panels in (**A**,**B**) with magnifications), which instead localize at the periphery of the well. Pictures were taken after 72 h and are representative of 1 out of 3 reproducible experiments performed in quadruplicate (original magnification 2.5×). (**C**–**F**) Migration (**C**,**D**) and invasion (**E**,**F**), through a 10 µg/mL layer of Matrigel, of PC3 shCTR and PC3 shNLGN1 (**C**,**E**) or MIA PaCa-2 PLVX and MIA PaCa-2 PezNLGN1 (**D**,**F**) by XCELLigence. Histograms show the migration and invasion rate in terms of cell index at the experimental end point (24 h) and values are expressed as the mean ± SE of 3 independent experiments performed in quadruplicate. One-way ANOVA with Bonferroni test: *, *p* < 0.05 **, *p* < 0.01, ***, *p* < 0.001. (**G**,**H**) Invasion rate of PC3 shCTR (**G**) or MIA PaCa-2 PezNLGN1 (**H**) assessed by XCELLigence in absence or presence of a GFRα1-blocking antibody. Graphs show the invasion rate in terms of cell index in time (hours) and values are expressed as mean ± SE of 2 independent experiments performed in quadruplicate. One-way ANOVA with Bonferroni test: *, *p* < 0.05 **, *p* < 0.01, ***, *p* < 0.001. (**I**,**J**) Cell proliferation, assessed by the Cell Titer Glo assay, of PC3 shCTR and PC3 shNLGN1 (**I**) or MIA PaCa-2 PLVX and MIA PaCa-2 PezNLGN1 (**J**). Graphs show the luminescence as arbitrary units and values are expressed as mean ± SD of one representative experiment performed in triplicate. The results analyzed with one-way ANOVA with Bonferroni test were not statistically different.

To verify the existence of a role for GDNF in NLGN1-induced phenotypes, we performed a DRG invasion assay in presence of a GFRα1 receptor-blocking antibody. As visible in Figure 3A, while in the absence of receptor-blocking antibody PC3 cells physiologically expressing NLGN1 invaded the neurites (left panel with magnification), in the presence of antibody, PC3 cells remained stacked at the periphery of the well (right panel with magnification). MIA PaCa-2, overexpressing NLGN1, behave in the same manner (Figure 3B left and right panels). Hence, the anti-GFRα1, receptor-blocking antibody inhibited NLGN1-induced migration and invasion of the ganglion (Figure 3A,B). To further dissect the NLGN1–GDNF functional interaction, we performed in vitro assays to study cell migration and invasion through a porous membrane, using the XCelligence CIM (cellular invasion/migration) plates that allow monitoring cellular responses in real time (Figure 3C–H). In these assays, NLGN1 expression was modulated and GDNF stimulation was performed in conjunction with the soluble form of its co-receptor, GFRα1. Furthermore, the invasion assay was performed in the presence of a coating of the reconstituted basal membrane Matrigel, which contains laminin (Figure 3E,F). Laminin is one of the most abundant ECM molecules in the peripheral nervous system [12,13] and facilitates prostate cancer metastatization through the neural route [13]. The presence of NLGN1 was critical for the response of both MIA PaCa-2 and PC3 cells to GDNF/GFRα1 in both migration (Figure 3C,D) and invasion (Figure 3E,F). Indeed, if NLGN1 was silenced, PC3 cells did not migrate and lost their invasive capacity in response to GDNF/GFRα1 (Figure 3C,E). If NLGN1 was overexpressed, MIA PaCa-2 cells migrated and invaded at a faster rate in response to GDNF/GFRα1 (Figure 3D,F). Mimicking the effect on the DRG invasion, the GFRα1-blocking antibody strongly reduced invasion in response to the DRG-conditioned media of both cell types (Figure 3G,H). NLGN 1 modulation or GDNF/GFRα1 treatment did not affect the proliferative capacity of PC3 or MIA PaCa-2 cells (Figure 3I,J). Finally, we confirmed that only in the presence of NLGN1 was GDNF able to induce invasion in another pancreatic ductal adenocarcinoma cell line, PT45 (Figure S2B), which basally express NLGN1 (Figure S2A). As for the other cell types, NLGN1 presence or GDNF treatment did not affect cell growth of PT45 cells (Figure S2C).

### 3.4. NLGN1 Mediates the Activation of Cofilin and Affects Filopodia Dynamics in Response to GDNF in Cancer Cells

To gain some insights into the molecular mechanism of the GDNF–NLGN1 activities, we evaluated the role of cofilin, the F-actin-severing protein required for cytoskeleton reorganization and filopodia formation, which drives cell migration. Cofilin drives invasion and metastasis in response to TGF-beta in prostate cancer [14] and, very interestingly, NLGN1 has been shown to modulate long-term potentiation (LTP) in hippocampal neurons through cofilin activity modulation [15].

**Figure 4:**
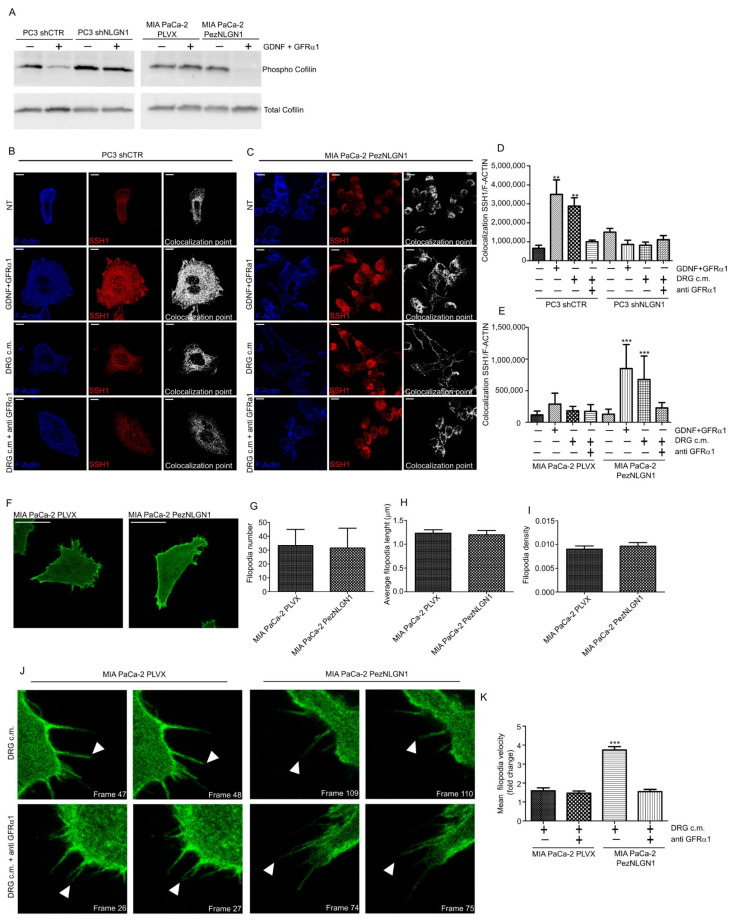
NLGN1 is necessary for cofilin activation by GDNF/GFRα1 treatment. (**A**) Western blotting analysis of Cofilin phosphorylation state on PC3 shCTR and PC3 shNLGN1 or MIA PaCa-2 PLVX and MIA PaCa-2 PezNLGN1 cells treated with 100ng/mL GDNF + 400 ng/mL GFRα1 for 5 min. Images are representative of 1 out of 3 reproducible experiments. Graphs show the densitometry of phospho-Cofilin, normalized on total cofilin. Fold change is calculated with respect to PC3 shCTR and Mia PaCa PLVX cells and values are expressed as mean ± SE (*n* = 3 independent experiments). (**B**–**E**) Confocal analysis of SSH1 (red) and F-Actin 647 (Blue) colocalization upon NLGN1 modulation on PC3 shCTR and PC3 shNLGN1 (**B**,**D**) or MIA PaCa-2 PLVX and MIA PaCa-2 PezNLGN1 (**C**,**E**) treated with 100ng/mL GDNF + 400 ng/mL GFRα1, DRG c.m or DRG c.m. + anti GFRα1 antibody for 5 min. The images are representative of 1 out of 3 reproducible experiments. Scale bar: 10 µm. The graphs in (**C**,**D**) show the colocalization rate between SSH1/F-actin measured at the cortical level as mean ± SE (*n* = 3, total cells analyzed *n* = 10). One-way ANOVA with Bonferroni test: **, *p* < 0.01, ***, *p* < 0.001. (**F**–**I**) Filopodia physical parameters of MIA PaCa-2 PLVX and MIA PaCa-2 PezNLGN1 cells, transiently transfected with LifeAct-pEGFPN1. Images in (**F**) are representative of 1 out of 3 reproducible experiments. Scale bar: -. Filopodia number (**G**), length (**H**), and density (number/cell edge, (**I**)) were detected using the Fiji ImageJ plugin FiloQuant. Values are expressed as mean ± S.E (*n* = 3, total cells analyzed *n* = 9), The results analyzed with one-way ANOVA with Bonferroni test were not statistically different. (**J**–**L**) Filopodia dynamics, upon a nerve input, of MIA PaCa-2 PLVX and MIA PaCa-2 PezNLGN1 cells, transiently transfected with LifeAct-pEGFPN1 and treated with DRG c.m. or DRG c.m. + an anti GFRα1 antibody. Cells were acquired using a Leica TCS SP8 AOBS (63× objective) for 5 min (one picture every 2.5 s). Filopodia were manually tracked using TrackMate (ImageJ-based tracking tool). Images in (**J**) are representative of 1 out of 3 reproducible experiments and show the filopodia movements (white arrowheads) frame to frame. Graph in (**K**) is the quantification of mean filopodia tip velocity (**L**) frame to frame represented as fold change versus MIA PaCa-2 PLVX cells treated with only the DRG-conditioned medium (c.m.). Values are expressed as mean ± SE (*n* = 3, total cells analyzed *n* = 11 up to 14). One-way ANOVA with Bonferroni test: ***, *p* < 0.001.

We first performed Western blotting analysis of inactive/active (i.e., respectively, phosphorylated/dephosphorylated) cofilin, showing that in the presence of NLGN1, GDNF/GFRα1 sharply activated cofilin in PC3 and MIA PaCa-2 cells, while in the absence of NLGN1, GDNF/GFRα1 showed no effect (Figure 4A). Cofilin is inactivated by phosphorylation by LIM-kinases and reactivated by dephosphorylation by slingshot (SSH1) phosphatase [16,17]. Since cofilin–phosphatase activity of SSH1 is increased by F-actin binding [18], we studied SSH1/F-actin colocalization in PC3, MIA PaCa-2, and PT45 cells (Figure 4B–E and Figure S3). When NLGN1 is expressed, SSH1/F-actin colocalization is significantly increased in the cortical region of the cytosol (Figure S4). The use of the GFRα-blocking antibody reversed this behavior (Figure 4B–E). These data indicate that the NLGN1/cofilin/SSH1 axis may play a crucial role in actin depolymerizing activity and in cell motility in response to a neural input. To investigate this aspect, we analyzed the actin filopodia dynamics in Mia PaCa-2 cells (Figure 4F) and we found that, although no changes were observed in terms of filopodia number (Figure 4G), length (Figure 4H), or density (Figure 4I), the presence of NLGN1 changed filopodia dynamics in terms of movement velocity (Figure 4J,K, and supplementary videos V1–4).

## 4. Conclusions

Our study provides the first widespread analysis of NLGN1 expression in prostatic and pancreatic cancer at the tissue level. The expression pattern in the prostate suggests that the expression of this protein is present during the mildly aggressive phases of progression, and falls at higher stages, which is in agreement with the low expression of NLGN1 in pancreatic cancer at all stages that we analyzed. Probably, the highly aggressive nature and rapid progression of pancreatic cancer, and the paucity of surgical samples at low stages, makes it difficult to isolate the window of expression of NLGN1 in this disease. On the other hand, it is very interesting that the expression of NLGN1 is retained in the metastatic biopsies. Globally, our results further prove the existence of molecular and functional crosstalk between the nervous system and cancer, contributing to the emerging field of cancer neuroscience [19]. NLGN1 was shown here to function along one of the most represented and pleiotropic nervous factors in the nerve microenvironment [7], possibly opening new therapeutic avenues. Furthermore, in a highly interesting parallel with the central nervous system, cofilin appears to mediate NLGN1′s role in both synaptic plasticity [15] and cancer cell movement. Finally, we show here that NGLN1 affects the migratory abilities of cells rather than their proliferation, an important validation of our results in the vascular system [4].

## Supplementary Materials

The following supporting information can be downloaded at: https://www.mdpi.com/article/10.3390/cells11020280/s1. These include Supplementary material and methods, Supplementary figures S1, S2, S3, S4. Supplementary videos V1–V4.
