# Supplementary material for: Tumoral Neuroligin 1 Promotes Cancer–Nerve Interactions and Synergizes with the Glial Cell Line-Derived Neurotrophic Factor
Source: Cells. 2022 Jan 14;11(2):280. doi: 10.3390/cells11020280 (PMC8774081; doi:10.3390/cells11020280)
Supplement: Supplementary file 1 [file cells-11-00280-s001.zip › cells-1544550-supplementary.pdf]

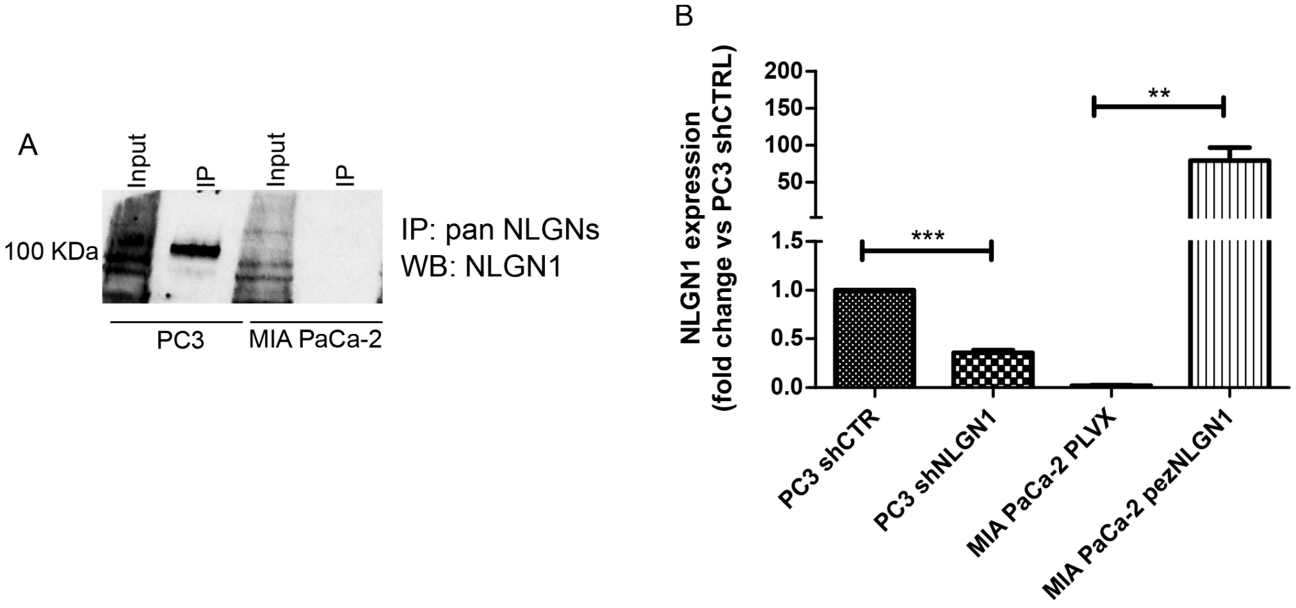

Figure S1: NLGN1 expression and modulation in the prostatic and pancreatic cell lines used in this paper. (A) Immunoprecipitation assay was performed in PC3 and MIA PaCa-2 cells using an anti-pan-NLGNs (L067) antibody, and immunoblotting was conducted with a monoclonal antibody (4C12) able to recognize NLGN1. The image shown is representative of 1 out of 3 reproducible experiments. (B) qRT–PCR of NLGN1 expression level on PC3 cells transduced with lentiviral vector containing a control short hairpin RNA (shCTR) or with one specific short hairpin RNA sequence targeting NLGN1 (shNLGN1), and MIA PaCa-2 cells transduced the lentiviral vector PLVX empty (PLVX) or with a lentiviral vector containing the human cDNA sequence of NLGN1 (PezNLGN1). Fold change is calculated with respect to PC3 cells transfected with shRNA CTR and values are expressed as mean ± SEM (n = 10 independent experiments). Student *t*-test, two tailed PC3 shCTR vs. shNLGN1: ***, $p < 0.001$; MIA PaCa-2 PLVX vs. Pez NLGN1: **, $p < 0.01$.

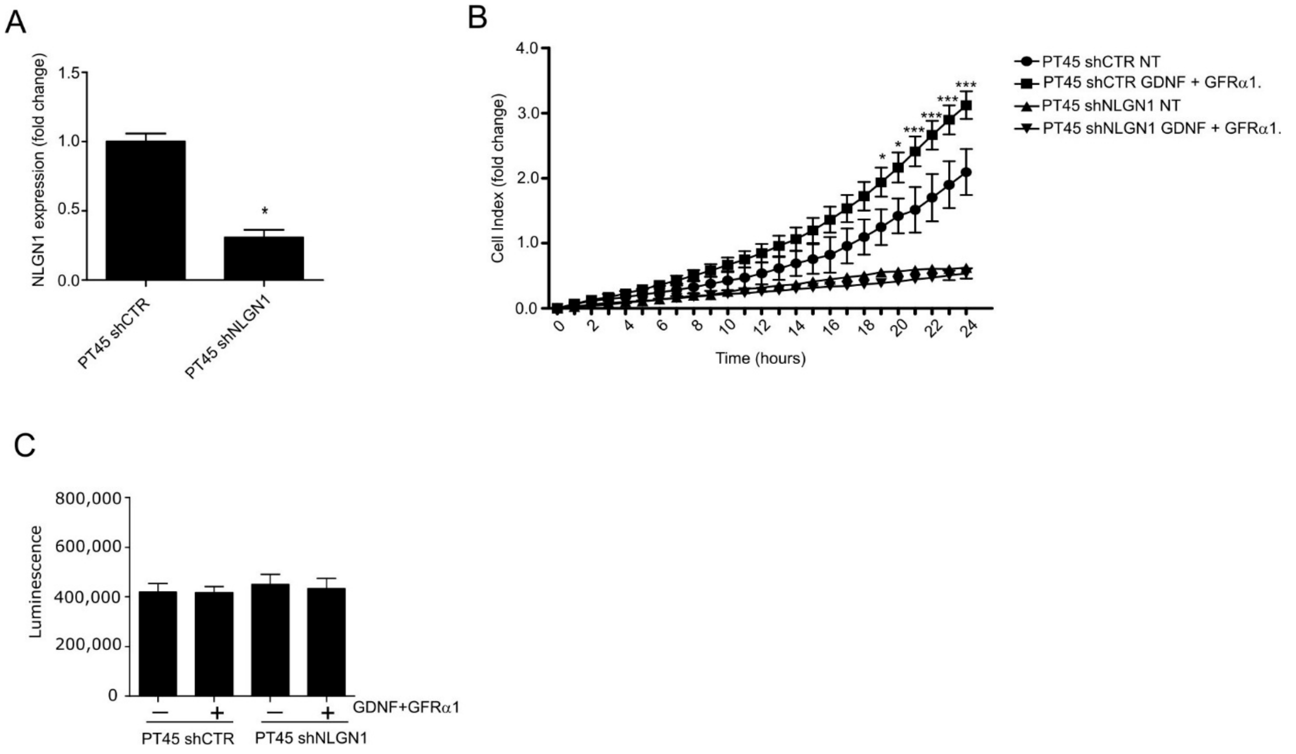

Figure S2: NLGN1 modulation affects cell invasion but not proliferation in the pancreatic cell line PT45. (A) qRT–PCR of NLGN1 expression level on PT45 cells transduced with lentiviral vector containing a control short hairpin

RNA (shCTR) or with one specific short hairpin RNA sequence targeting NLGN1 (shNLGN1). Fold change is calculated with respect to PT45 cells transfected with shRNA CTR and values are expressed as mean ± SEM (n = 4 independent experiments). Student *t*-test two tailed PC3 shCTR vs. shNLGN1: *, $p < 0.05$. B) invasion rate, through a 10 μg/ml layer of Matrigel, of PT45 shCTR and PT45 shNLGN1 cells in presence or absence of GDNF (100 ng/ml) + GFRα1 (400 ng/ml) by XCELLigence. Graph show the invasion rate in terms of cell index over time (hours) and values are expressed as mean ± SD of 1 out of 3 independent experiments performed in quadruplicate. One-way ANOVA with Bonferroni test: *, $p < 0.05$, ***, $p < 0.001$. (C) Cell proliferation, assessed by the Cell Titer Glo assay, of PT45 shCTR and PT45 shNLGN1. Histogram shows the luminescence 72 hours as arbitrary units and values are expressed as mean ± SD of 1 representative experiment performed in triplicate. The results analyzed with one-way ANOVA with Bonferroni test were not statistically different.

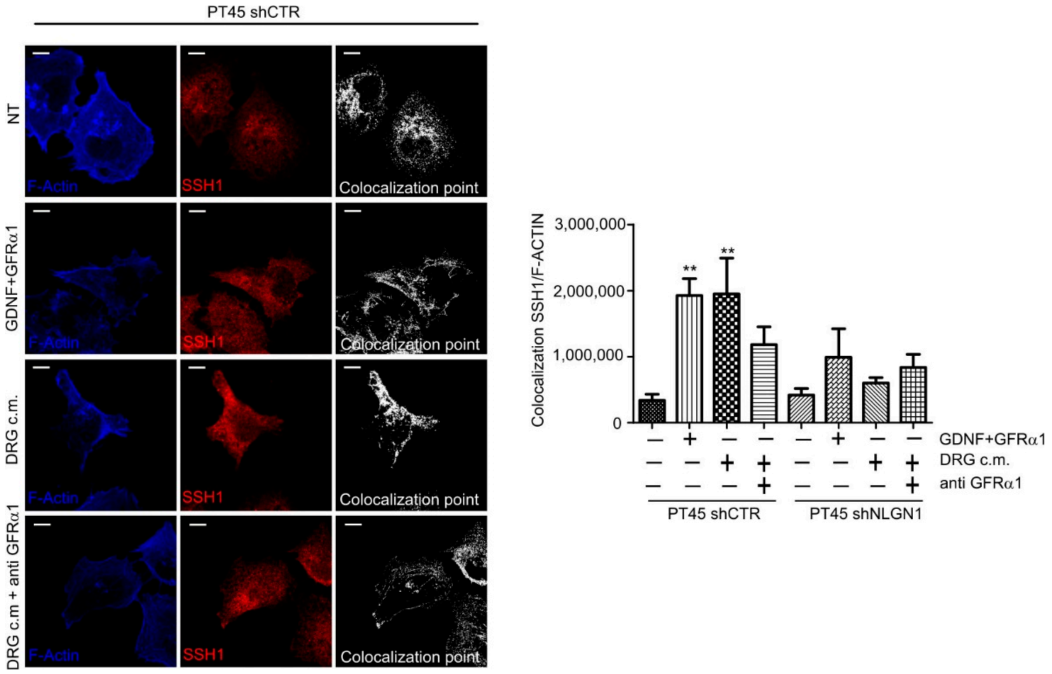

Figure S3: Colocalization of SSH1 and F-actin in the cortical region of PT45 cells. Confocal analysis of SSH1 (red) and F-Actin 647 (Blue) colocalization upon NLGN1 modulation on PT45 shCTR and PT45 shNLGN1 treated with 100 ng/ml GDNF + 400 ng/ml GFRα1, DRG c.m. or DRG c.m. + anti GFRα1 antibody for 5 minutes. Scale bar: 10 μm. The graph shows the colocalization rate, as arbitrary unit, between SSH1/F-actin measured at the cortical level. Values are expressed as mean ± SD (n = 6), One-way ANOVA with Bonferroni test: **, $p < 0.01$.

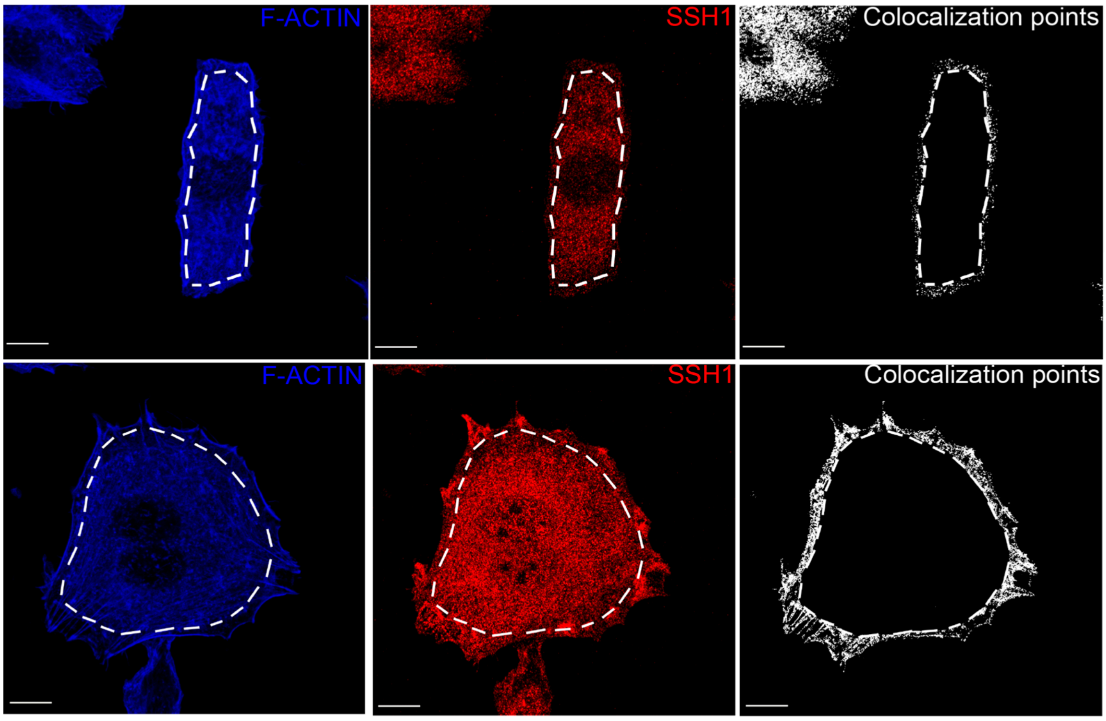

Figure S4: SSH1 cortical signal quantification. Confocal microscopy highlights the differences in terms of SSH1 (red) F-actin (blue) colocalization at the cell cortical level manually identified through the line tool of ImageJ (here indicated by the dashed line). Colocalization was quantified through the colocalization plugin of ImageJ (colocalize point panel). Scale bar: 10 μm.

Video1-4: NLGN1 enhances filopodia dynamics upon dorsal root ganglia (DRG)-conditioned media (c.m.) stimulation: MIA PaCa-2 PLVX (video 1 and 2) and MIA PaCa-2 PezNLGN1 (video 3 and 4) cells were transiently transfected with LifeAct-pEGFPN1 and treated with DRG c.m. (video 1 and 3) or DRG c.m. + an anti GFRα1 antibody (video 2 and 4). Cells were acquired using a Leica TCS SP8 AOBS (63x objective) for 5 min (one picture every 2.5 sec).

Supplementary Materials and Methods

Cell Lines

In this study, we used the prostate PC3 adenocarcinoma cell line, which was originally established from skeletal metastases, and the pancreatic ductal adenocarcinoma cell lines MIA PaCa-2 and PT45.

Animals

Mice C57BL/6 and NOD/SCID (all males) were maintained in the animal facility of the Institute for Cancer Research (IRCC) under standard housing conditions. All animal experiments were performed in compliance with the ARRIVE guidelines and carried out in accordance with EU Directive 2010/63/EU (Italian D.L. 4 March 2014, n. 26).

RNA Extraction, Retrotranscription, and qRT–PCR

The total RNA was extracted using TRIzol (Invitrogen) according to the manufacturer's instructions. First-strand cDNA was generated from 1 μg of total RNA using the High-Capacity cDNA Reverse Transcription Kit (Invitrogen). mRNA expression was analyzed by quantitative real-time reverse transcription–PCR (qRT–PCR) using the TaqMan gene expression assay (human NLGN1, Hs00208784_m1). The mRNA levels, analyzed in triplicate, were normalized by using as housekeeping genes, the human (Hs00427620_m1) TATA-binding box protein, and determined with the formula $2^{-\Delta\Delta CT}$.

Lentiviral Infection

The PLVX (pLVX-puro expression vector), PezNLGN1 (pez-lv 105 expression vector) and shCTR and shNLGN1 (pGFP-C-shLenti shRNA-29 expression vector, Origene) lentivirus were produced as described (*Bottos A., Destro E., Rissone A., Graziano S., Cordara G., Assenzio B., Cera M. R., Mascia L., Bussolino F., Arese M. (2009) The synaptic proteins neurexins and neuroligins are widely expressed in the vascular system and contribute to its functions. Proc. Natl. Acad. Sci. U.S.A. 106, 20782–20787)*. Cells were seeded at concentrations of 2 × 10^5 cells/ml in a 10 cm/diameter tissue culture dish, transduced for 36 h with lentiviral particles in the presence of 8 μg/ml of Polybrene (Sigma) and selected for 48 h with 2 mg/ml of puromycin. The efficiency of cell infection was determined by qRTPCR analysis.

Cell Proliferation

Cells were seeded at the concentration of 3000 cells/well in 96-well plates in RPMI-1640 10% FBS in presence of absence of 100 ng/ml GDNF + 400 ng/ml GFRα1. Cell proliferation was measured at 0, 24, 48, 72, and 96 hours by Cell Titer Glo Luminescent Assay kit (G7572, Promega).

Immunoprecipitation and Immunoblotting Analysis

Subconfluent cancer cells were homogenized in cold EB buffer (10 mM Tris-HCl, pH 7.5, 150 mM NaCl, 5 mM EDTA, pH 8; 10% glycerol, 1% Triton X-100, protease and phosphatase inhibitors, 50 μg/ml of pepstatin, 50 μg/ml of leupeptin, 10 μg/ml of aprotinin, 1 mM PMSF, 100 μM ZnCl2, 1 mM sodium orthovanadate, and 10 mM NaF). After centrifugation (20 min, 4 °C at 10,000 × *g*), the supernatants were quantified with the BCA Protein Assay Reagent Kit (Pierce Chemical Co.). Each sample was then incubated overnight at 4 °C with rabbit polyclonal anti-NLGNs antibody (L067), used at a final concentration of 2.5 μg/mg of protein. The immune complexes were recovered on protein A-Sepharose for 1 h and 30 min, pelleted, and washed 4 times with lysis buffer. The proteins were separated by 10% SDS-PAGE electrophoresis gel (Biorad), transferred to polyvinylidene difluoride membrane (PVDF, Biorad), and incubated with anti-mouse monoclonal anti-NLGN1 antibody (4C12 1:1000). For Western blotting analysis, 30 μg of total protein, lysed in cold buffer consisting of 20 mM Tris (pH 7.5), 150 mM NaCl, 1% Triton X-100, 2× complete protease inhibitors, 17.5 mM β-glycerophosphate, 20 mM NaF, 1 mM sodium orthovanadate, and 500 μM E64, were separated by 4-12% SDS-PAGE electrophoresis gel, transferred to a nitrocellulose membrane (Biorad), and then immunodecorated with specific antibodies against phospho-Cofilin and total Cofilin. For immunoprecipitation, proteins for each sample were pre-cleared by a 1 h incubation with A-Sepharose protein (Amersham Biosciences). Secondary antibodies were HRP-conjugated goat anti-rabbit or anti-mouse (Jackson ImmunoResearch). Antibodies were detected with ECL reagent (Amersham/GE Healthcare).

Pancreatic and Prostate Cancer Tissue Microarrays (TMAs)

NLGN1 expression was evaluated by immunohistochemistry (IHC) on two TMAs of pancreatic cancers (PA961f and PA2081c—Biomax) and one TMA of prostate adenocarcinoma (PA961f—Biomax). Hematoxylin and eosin (H&E) sections were available online for morphology evaluation.

The immunostaining was performed using an automated immunostainer (Ventana BenchMark Ultra Auto-Stainer, Roche Ltd). Endogenous peroxidase activity was blocked by 20 min of incubation with 0.3% hydrogen peroxidase. Slides were tested using antibody anti-NLGN1 (Neuromab Clone N97A/31), 1/100 dilution. Sections were tested with a streptavidin-biotin-peroxidase kit (UltraVision Large Volume Detection System Anti-Polyvalent, HRP, LabVision, USA), and after incubation, the reaction product was detected using diaminobenzidine (DAB). Finally, the sections were counterstained with Mayer's Hematoxylin and mounted with aqueous mounting medium. Nerve tissue was used as internal control. NLGN1 expression was scored as present (1, 2, 3) or absent (0).

In Vitro Migration and Invasion

Real-time cell analysis (RTCA) of cell migration and invasion was monitored using a CIM (cellular invasion/migration) plate and xCELLigence DP System (Acea Bioscience, Inc.) according to the manufacturer's instructions. Cells were serum-starved overnight in RPMI-1640 with 2% FBS. Before cell seeding (5×10^4 cells/well), the upper chamber was filled with 30 μl of RPMI-1640 w/o FBS, and the lower chamber was filled with 160 μl of RPMI-1640 10% FBS in presence or absence of 100 ng/ml GDNF + 400 ng/ml GFRα1 or DRG-conditioned medium in presence or absence of a GFRα1-blocking antibody (10 μg/ml). For the invasion assay, the electrodes were previously coated with 10% (v/v) growth factor-reduced Matrigel matrix (Corning). Cell index was monitored every 10 minutes for 24 hours.

Immunofluorescence and Live Imaging

Cells, treated as indicated in the results section, plated on glass coverslips, were fixed with 4% PFA for 10 min at room temperature (RT), permeabilized with 0.1% Triton–X in PBS for 5 min at RT, and saturated with 10% goat serum/1% BSA/0.1% Tween-20/0.3M glycine in PBS for 1 h at RT. Slides were incubated with the primary antibodies rabbit polyclonal anti-SSH1 (1:200, Abcam) in 1% BSA PBS overnight at 4°C and Phalloidin 647 (ThermoFisher) in 0.1% Tween-20 in PBS for 30 min at RT. The secondary antibody (green) was Alexa Fluor® 555 goat anti-rabbit IgG (H+L) used at a 1/500 dilution for 1 h at RT.

Immunofluorescence images were taken with the Leica TCS SP8 microscope. Colocalization analysis was performed using the colocalization plugin of ImageJ. Image acquisition was performed by adopting a laser power, gain, and offset settings that allowed maintaining pixel intensities (gray scale) within the 0–255 range and hence avoid saturation.

For live imaging, MIA PaCa-2 cells, plated in a μ-slide 8-well IbiTreat, were transiently transfected with the fluorescent-tagged cDNA pEGFPN1 LifeAct (courtesy of Prof. Guido Serini laboratory). Cells were analyzed by using a Leica TCS SP8 AOBS confocal microscope equipped with two HyD, PL APO 63×/1.4. NA immersion objective was employed. 512 × 512-pixel images were acquired at pixel size = 161.51–182.01 nm. Image acquisition was performed by adopting a laser power, gain, and offset settings that allowed maintaining pixel intensities (gray scale) within the 0–255 range and hence avoid saturation. Movies to image the actin filopodia were acquired for 5 min, taking 1 frame every 2.5 s. In particular, we quantified the physical parameters (number, length, and density) of filopodia using the Fiji plugin Filoquant as described *(Jacquemet, G., Paatero, I., Carisey, A.F., Padzik, A., Orange, J.S., Hamidi, H., and Ivaska, J. (2017). FiloQuant reveals increased filopodia density during breast cancer progression. J. Cell Biol. 216, 3387–3403).*

Filopodia dynamics, in terms of velocity and tip distance, were manually tracked using Fiji plugin TrackMate. We tracked from 5 to 10 filopodia/cell over the filopodia lifetime in at least 11 cells (up to 14).
